# Thyroid Hormone Levels and Its Co-relation With Human Chorionic Gonadotropin in Patients With Molar Pregnancy

**DOI:** 10.7759/cureus.114817

**Published:** 2026-08-19

**Authors:** Priyadarsini Singh, Swayamprava Behera, Lipsa Patra, Sasmita Behuria, Pournava Pratyasa Pradhan, Roopa Parida

**Affiliations:** 1 Obstetrics and Gynaecology, Government Medical College and Hospital, Phulbani, IND; 2 Obstetrics and Gynaecology, Maharaja Jajati Keshari Medical College and Hospital, Jajpur, IND; 3 Obstetrics and Gynaecology, Srirama Chandra Bhanja (SCB) Medical College and Hospital, Cuttack, IND; 4 Obstetrics and Gynaecology, Postgraduate Institute of Medical Education and Research (PGIMER) and Capital Hospital, Bhubaneswar, IND; 5 Obstetrics and Gynaecology, All India Institute of Medical Sciences, Bhubaneswar, IND; 6 Clinical Pharmacology, Seth Gordhandas Sunderdas Medical College (GSMC) and King Edward Memorial (KEM) Hospital, Mumbai, IND

**Keywords:** gestational trophoblastic disease, human chorionic gonadotropin, hydatidiform mole, hyperthyroidism, thyroid hormones

## Abstract

Introduction

Gestational trophoblastic disease (GTD) is characterized by abnormal trophoblastic proliferation, with hydatidiform mole being its most common form. Markedly elevated serum human chorionic gonadotropin (hCG) concentrations in molar pregnancy can stimulate thyroid-stimulating hormone receptors, resulting in thyroid dysfunction. This study aimed to evaluate thyroid hormone levels and their correlation with serum β-human chorionic gonadotropin (β-hCG) concentrations in patients with molar pregnancy and to compare thyroid function between complete and partial hydatidiform mole.

Materials and methods

This prospective observational study was conducted in the Department of Obstetrics and Gynaecology, Srirama Chandra Bhanja (SCB) Medical College and Hospital, Cuttack, Odisha, India, from January 2022 to December 2023. Eighty-eight women with histopathologically confirmed hydatidiform mole were included after excluding 26 patients who had received initial treatment elsewhere and were referred exclusively for oncological management. Serum β-hCG, thyroid-stimulating hormone (TSH), free triiodothyronine (free T3), free thyroxine (free T4), total triiodothyronine (total T3), and total thyroxine (total T4) were measured before uterine evacuation. Thyroid hormone profiles were compared between complete and partial mole, and correlations between serum β-hCG and thyroid hormone parameters were analyzed using Pearson's correlation coefficient.

Results

Among 15,844 deliveries, 88 women with histopathologically confirmed hydatidiform mole were analyzed, yielding a hospital-based frequency of 5.5 per 1,000 deliveries. Complete mole constituted 57 (64.8%) cases and partial mole 31 (35.2%). Patients with complete mole had significantly lower TSH (0.82±0.71 vs. 1.96±0.82 µIU/mL; p<0.001) and significantly higher free T3, free T4, total T3, total T4, and β-hCG levels than those with partial mole (all p<0.001). Serum β-hCG demonstrated a significant negative correlation with TSH (r=−0.61; p<0.001) and positive correlations with free T3 (r=0.73), free T4 (r=0.69), total T3 (r=0.65), and total T4 (r=0.67) (all p<0.001).

Conclusion

Complete hydatidiform mole was associated with significantly higher serum β-hCG concentrations and more pronounced thyroid dysfunction than partial hydatidiform mole. Serum β-hCG concentrations demonstrated a significant negative correlation with TSH and positive correlations with free and total T3 and T4 levels, supporting a close association between trophoblastic activity and thyroid function. These findings suggest that thyroid dysfunction may be particularly relevant in women with complete hydatidiform mole or markedly elevated serum β-hCG concentrations; however, larger multicentric studies with adjustment for potential confounding factors and longitudinal follow-up are warranted to clarify the clinical significance of these associations.

## Introduction

Gestational trophoblastic disease (GTD) comprises a heterogeneous group of pregnancy-related disorders characterized by abnormal proliferation of trophoblastic tissue [1]. It encompasses a spectrum ranging from benign hydatidiform mole (complete and partial mole) to malignant gestational trophoblastic neoplasia (GTN), which includes invasive mole, choriocarcinoma, placental site trophoblastic tumor, and epithelioid trophoblastic tumor [2]. Although GTD is relatively uncommon, it is of considerable clinical importance because of its potential for local invasion and distant metastasis, while early diagnosis and appropriate management are associated with an excellent prognosis [3]. Hydatidiform mole represents the most common form of GTD and is the precursor lesion for the majority of cases of GTN [4].

The incidence of GTD varies widely across different geographic regions and ethnic populations [4]. Higher incidences have been reported from several Asian countries compared with Western populations, highlighting the influence of genetic, nutritional, and socioeconomic factors [5,6]. In India, available epidemiological data remain limited and are largely hospital-based, resulting in an underestimation of the true disease burden [7]. Early diagnosis of molar pregnancy is essential to prevent complications such as excessive uterine bleeding, hyperemesis gravidarum, pre-eclampsia, persistent trophoblastic disease, and progression to gestational trophoblastic neoplasia [8].

Human chorionic gonadotropin (hCG), a glycoprotein hormone secreted by syncytiotrophoblastic cells, plays a pivotal role in the diagnosis, monitoring, and follow-up of patients with molar pregnancy [9]. Structurally, hCG shares a common α-subunit with thyroid-stimulating hormone (TSH), luteinizing hormone (LH), and follicle-stimulating hormone (FSH), whereas its β-subunit confers hormonal specificity [10]. In molar pregnancy, markedly elevated hCG concentrations can exert thyrotropic activity because of structural similarities between hCG and TSH, allowing hCG to stimulate TSH receptors and resulting in increased synthesis and release of thyroid hormones with suppression of serum TSH levels [11,12]. Consequently, patients with complete hydatidiform mole are more likely to develop thyroid dysfunction than those with partial mole, particularly in the presence of markedly elevated hCG concentrations. For clinical interpretation, biochemical (subclinical) hyperthyroidism is characterized by a low or suppressed TSH concentration with free thyroid hormone levels within the reference range, whereas overt hyperthyroidism is characterized by a low or suppressed TSH concentration accompanied by elevated free T4 and/or free T3 levels.

Despite the well-recognized association between elevated serum β-hCG levels and thyroid dysfunction in molar pregnancy, studies evaluating the correlation between serum β-hCG concentrations and thyroid hormone profiles in the Indian population remain limited. A better understanding of this relationship may help identify the association between trophoblastic activity and thyroid function and provide clinically relevant information for the assessment of patients with molar pregnancy. Therefore, the present study aimed to evaluate thyroid hormone levels and their correlation with serum β-hCG concentrations in patients with molar pregnancy and to compare thyroid hormone profiles between complete and partial hydatidiform mole.

## Materials and methods

This prospective observational study was conducted in the Department of Obstetrics and Gynaecology, SCB Medical College and Hospital, Cuttack, Odisha, India, over a period of 24 months from January 2022 to December 2023. The study was approved by the Institutional Ethics Committee, SCB Medical College and Hospital, Cuttack, Odisha (IEC Application No. 845). Written informed consent was obtained from all participants before enrolment in the study.

All consecutive pregnant women diagnosed with hydatidiform mole during the study period were screened for eligibility. A total of 114 patients with GTD were identified during the study period. Of these, 26 patients who had received initial treatment elsewhere and were referred to the institution exclusively for oncological management were excluded. Women with ultrasonographically suspected complete or partial hydatidiform mole, histopathological confirmation, and written informed consent were eligible for inclusion. Additional exclusion criteria included prior treatment at another institution, referral exclusively for chemotherapy or oncological follow-up, known pre-existing thyroid disorders or current thyroid medication use, and incomplete clinical or laboratory records. A total of 88 patients fulfilled the eligibility criteria and were included in the final analysis.

Detailed demographic, obstetric, clinical, laboratory, radiological, and histopathological data were recorded using a predesigned case record form. Information collected included maternal age, gravidity, parity, gestational age at diagnosis, previous abortions, number of living children, and histopathological diagnosis as complete or partial hydatidiform mole.

All participants underwent ultrasonographic examination of the abdomen and pelvis to confirm the diagnosis of molar pregnancy before definitive treatment. Ultrasonographic findings assessed included the characteristic snowstorm appearance, hydropic changes within the uterine cavity, presence or absence of fetal tissue, bilateral theca lutein ovarian cysts, and any associated pelvic pathology [13].

Venous blood samples were collected before uterine evacuation for the estimation of serum β-human chorionic gonadotropin (β-hCG), thyroid-stimulating hormone (TSH), free triiodothyronine (free T3), free thyroxine (free T4), total triiodothyronine (total T3), and total thyroxine (total T4) [14]. All biochemical investigations were performed in the central laboratory of the institution using standardized laboratory procedures.

The primary outcome was the correlation between serum β-hCG concentrations and thyroid hormone parameters in patients with molar pregnancy. The secondary outcome was the comparison of thyroid hormone profiles between patients with complete and partial hydatidiform mole.

Data were entered into Microsoft Excel (Microsoft, Redmond, WA) and analyzed using IBM SPSS Statistics for Windows, Version 22.0 (IBM Corp., Armonk, NY, USA). Continuous variables were expressed as mean±standard deviation (SD), while categorical variables were presented as frequency and percentage (N (%)). Comparisons between complete and partial hydatidiform mole were performed using the independent Student's t-test for continuous variables. Categorical variables were analyzed using the chi-square test or Fisher's exact test, as appropriate. The correlation between serum β-hCG levels and thyroid hormone parameters was assessed using the Pearson correlation coefficient (r). All statistical tests were two-tailed, and a p-value <0.05 was considered statistically significant. No multivariable analysis was performed to adjust for potential confounding factors such as gestational age, maternal age, gravidity, or parity.

## Results

During the study period, a total of 15,844 deliveries occurred at the study institution. Among these, 114 patients were diagnosed with GTD. After excluding 26 patients who had received initial treatment elsewhere and were referred exclusively for oncological management, 88 patients with histopathologically confirmed hydatidiform mole were included in the final analysis. The hospital-based frequency of hydatidiform mole was 5.5 per 1,000 deliveries (Table 1).

**Table 1:** Hospital-Based Frequency of Hydatidiform Mole and Study Population Selection Data are presented as numbers or hospital-based frequency per 1,000 deliveries. The table summarizes the total number of deliveries, gestational trophoblastic disease (GTD) cases, excluded patients, final study population, and hospital-based frequency of hydatidiform mole.

Variable	Value
Total deliveries	15,844
Total GTD cases	114
Excluded (treated elsewhere)	26
Final study population	88
Hospital-based frequency of hydatidiform mole	5.5 per 1,000 deliveries

The mean maternal age of the study population was 26.8±5.4 years, with the majority of women belonging to the 25-29 years age group (31 (35.2%)), followed by the 20-24 years age group (24 (27.3%)). Most patients were multigravida (65 (73.9%)), while 23 (26.1%) were primigravida. The mean gravida and parity were 3.2±1.6 and 1.4±1.2, respectively. A history of previous abortion was present in 19 (21.6%) women. The mean gestational age at diagnosis was 11.4±2.7 weeks (Table 2).

**Table 2:** Baseline Demographic and Obstetric Characteristics of the Study Population (N=88) Continuous variables are presented as mean±standard deviation (SD), while categorical variables are presented as number (percentage) (n (%)). This table provides the baseline demographic and obstetric characteristics of women diagnosed with hydatidiform mole. Abbreviations: SD, Standard Deviation; N, Total number of study participants.

Variable	Value
Maternal age (years), mean±SD	26.8±5.4
Age <20 years	9 (10.2%)
20-24 years	24 (27.3%)
25-29 years	31 (35.2%)
30-34 years	16 (18.2%)
≥35 years	8 (9.1%)
Primigravida	23 (26.1%)
Multigravida	65 (73.9%)
Gravida, mean±SD	3.2 ± 1.6
Parity, mean±SD	1.4 ± 1.2
Previous abortions	19 (21.6%)
Living children	1.3 ± 1.1
Gestational age (weeks), mean±SD	11.4 ± 2.7

Of the 88 patients included in the study, 57 (64.8%) were diagnosed with complete hydatidiform mole, whereas 31 (35.2%) had partial hydatidiform mole, indicating that complete mole was the predominant histopathological subtype (Table 3).

**Table 3:** Distribution of Histopathological Types of Hydatidiform Mole Data are presented as number (percentage) (n (%)). The table shows the frequency distribution of complete and partial hydatidiform mole among the study population. Abbreviations: n, Number of patients.

Histopathology	n (%)
Complete mole	57 (64.8%)
Partial mole	31 (35.2%)
Total	88 (100%)

Patients with complete hydatidiform mole had a significantly higher mean gravida than those with partial mole (3.9±1.7 vs. 2.0±0.6; t=6.91, p<0.001). Similarly, the mean parity was significantly greater among women with complete mole (1.8±1.3 vs. 0.8±0.7; t=4.16, p<0.001). Women with complete mole also presented at a slightly higher gestational age (11.8±2.5 vs. 10.7±2.4 weeks; t=2.03, p=0.047). Although the mean maternal age was higher in the complete mole group (27.4±5.5 vs. 25.8±5.1 years) and previous abortions were more frequent (15 (26.3%) vs. 4 (12.9%)), these differences were not statistically significant (p>0.05) (Table 4).

**Table 4:** Comparison of Baseline Characteristics Between Complete and Partial Mole Continuous variables are presented as mean±standard deviation (SD), and categorical variables as number (percentage) (n (%)). Comparisons between groups were performed using the independent Student's t-test for continuous variables and the chi-square (χ²) test for categorical variables. A p-value <0.05 was considered statistically significant. Abbreviations: SD, Standard deviation; χ², chi-square test; t, Student's t-test.

Variable	Complete Mole (n=57)	Partial Mole (n=31)	Statistical Test (Value)	p-value
Age (years)	27.4±5.5	25.8±5.1	t=1.35	0.182
Gravida	3.9±1.7	2.0±0.6	t=6.91	<0.001
Parity	1.8±1.3	0.8±0.7	t=4.16	<0.001
Gestational age (weeks)	11.8±2.5	10.7±2.4	t=2.03	0.047
Previous abortions	15 (26.3%)	4 (12.9%)	χ²=2.11	0.147

The mean serum TSH level of the study population was 1.22±1.46 µIU/mL, while the mean free T3 and free T4 levels were 6.32±2.82 pg/mL and 1.91±0.86 ng/dL, respectively. The mean total T3 and total T4 concentrations were 2.48±0.83 ng/mL and 12.82±5.10 µg/dL, respectively. The mean serum β-hCG concentration was 356,722±194,744 mIU/mL, with values ranging from 39,647 to 944,793 mIU/mL (Table 5).

**Table 5:** Thyroid Hormone Profile and Serum β-hCG Levels of the Study Population Continuous variables are presented as minimum, maximum, and mean±standard deviation (SD). This table summarizes thyroid function parameters and serum β-human chorionic gonadotropin (β-hCG) concentrations in women with hydatidiform mole. Abbreviations: TSH, Thyroid-stimulating hormone; T3, triiodothyronine; T4, thyroxine; β-hCG, beta-human chorionic gonadotropin; SD, standard deviation.

Variable	Minimum	Maximum	Mean±SD
TSH (µIU/mL)	0.01	5.10	1.22±1.46
Free T3 (pg/mL)	2.5	13.5	6.32±2.82
Free T4 (ng/dL)	0.8	4.2	1.91±0.86
Total T3 (ng/mL)	1.1	4.8	2.48±0.83
Total T4 (µg/dL)	7.3	26.2	12.82±5.10
β-hCG (mIU/mL)	39,647	944,793	356,722±194,744

Patients with complete hydatidiform mole demonstrated significantly lower serum TSH levels than those with partial mole (0.82±0.71 vs. 1.96±0.82 µIU/mL; t=6.63, p<0.001). In contrast, free T3 (7.12±2.90 vs. 4.72±1.71 pg/mL; t=4.67, p<0.001), free T4 (2.34±0.74 vs. 1.24±0.39 ng/dL; t=8.41, p<0.001), total T3 (2.82±0.68 vs. 1.86±0.44 ng/mL; t=7.08, p<0.001), and total T4 (15.72±4.82 vs. 9.11±2.76 µg/dL; t=7.19, p<0.001) were significantly higher in the complete mole group. Mean serum β-hCG levels were also markedly elevated among patients with complete mole compared with partial mole (451,860±176,540 vs. 181,430±79,620 mIU/mL; t=8.96, p<0.001) (Table 6).

**Table 6:** Comparison of Thyroid Hormone Levels and Serum β-hCG Between Complete and Partial Hydatidiform Mole Continuous variables are presented as mean±standard deviation (SD). Comparisons between complete and partial mole were performed using the independent Student's t-test. A p-value <0.05 was considered statistically significant. Abbreviations: TSH, Thyroid-stimulating hormone; T3, triiodothyronine; T4, thyroxine; β-hCG, beta-human chorionic gonadotropin; SD, standard deviation; t, Student's t-test.

Variable	Complete Mole	Partial Mole	Statistical Test (Value)	p-value
TSH (µIU/mL)	0.82±0.71	1.96±0.82	t=6.63	<0.001
Free T3 (pg/mL)	7.12±2.90	4.72±1.71	t=4.67	<0.001
Free T4 (ng/dL)	2.34±0.74	1.24±0.39	t=8.41	<0.001
Total T3 (ng/mL)	2.82±0.68	1.86±0.44	t=7.08	<0.001
Total T4 (µg/dL)	15.72±4.82	9.11±2.76	t=7.19	<0.001
β-hCG (mIU/mL)	451860±176540	181430±79620	t=8.96	<0.001

Correlation analysis demonstrated a significant inverse relationship between serum β-hCG and TSH levels (r=-0.61, p<0.001). Conversely, β-hCG showed significant positive correlations with free T3 (r=0.73, p<0.001), free T4 (r=0.69, p<0.001), total T3 (r=0.65, p<0.001), and total T4 (r=0.67, p<0.001), indicating that higher serum β-hCG concentrations were associated with lower TSH and higher thyroid hormone levels (Table 7).

**Table 7:** Correlation Between Serum β-hCG Concentration and Thyroid Hormone Parameters Correlation coefficients are presented as Pearson's correlation coefficient (r). The relationship between serum β-hCG and thyroid hormone parameters was evaluated using Pearson's correlation analysis. A p-value <0.05 was considered statistically significant. Abbreviations: β-hCG, Beta-human chorionic gonadotropin; TSH, thyroid-stimulating hormone; T3, triiodothyronine; T4, thyroxine; r, Pearson's correlation coefficient.

Variable	Correlation (r)	p-value
TSH	-0.61	<0.001
Free T3	0.73	<0.001
Free T4	0.69	<0.001
Total T3	0.65	<0.001
Total T4	0.67	<0.001

## Discussion

The present study demonstrated that complete hydatidiform mole accounted for 57 (64.8%) cases, whereas 31 (35.2%) had partial hydatidiform mole. Women with complete mole also had significantly higher gravidity (3.9±1.7 vs. 2.0±0.6; p<0.001) and parity (1.8±1.3 vs. 0.8±0.7; p<0.001) than those with partial mole. Women with complete mole also had a slightly higher gestational age at diagnosis than those with partial mole (11.8±2.5 vs. 10.7±2.4 weeks; p=0.047). These findings are comparable to those reported by Düğeroğlu et al., who observed that patients with complete hydatidiform mole were older, had significantly higher parity, and exhibited more severe thyroid dysfunction than women with partial mole [12]. Similarly, Soper reported that complete hydatidiform mole represents the predominant subtype of gestational trophoblastic disease and is associated with higher β-hCG concentrations and a greater risk of systemic complications than partial mole [14].

The present study also demonstrated significantly lower serum TSH concentrations (0.82±0.71 vs. 1.96±0.82 µIU/mL; p<0.001) and significantly higher free T3 (7.12±2.90 vs. 4.72±1.71 pg/mL), free T4 (2.34±0.74 vs. 1.24±0.39 ng/dL), total T3 (2.82±0.68 vs. 1.86±0.44 ng/mL), total T4 (15.72±4.82 vs. 9.11±2.76 µg/dL), and serum β-hCG levels (451,860±176,540 vs. 181,430±79,620 mIU/mL) among women with complete mole (all p<0.001). These observations closely parallel those of Düğeroğlu et al., who reported significantly lower TSH together with significantly higher free T4 and total T4 concentrations in patients with complete hydatidiform mole than in those with partial mole [12]. Their study further demonstrated that thyroid dysfunction became more pronounced with increasing β-hCG levels, mole size, maternal age, and parity. Similar findings were reported by Badlaeva et al., who found significantly higher serum T3, T4, and free thyroxine index values among patients with hyperthyroid molar pregnancy compared with euthyroid patients [15]. The higher thyroid hormone concentrations observed in the complete mole group are biologically consistent with the greater trophoblastic burden and higher circulating β-hCG concentrations characteristic of complete mole.

An important finding of the present study was the significant inverse correlation between serum β-hCG and TSH (r=−0.61; p<0.001) and positive correlations with free T3 (r=0.73), free T4 (r=0.69), total T3 (r=0.65), and total T4 (r=0.67) (all p<0.001). These findings support the thyrotropic activity of hCG and the close association between trophoblastic activity and thyroid function. Zheng et al. similarly demonstrated significant positive correlations between serum β-hCG and T3, T4, and free thyroxine index, supporting an association between increasing hCG concentrations and thyroid stimulation [16]. Earlier work by Badlaeva et al. also showed that patients with molar pregnancy frequently exhibit elevated thyroid hormone concentrations in association with markedly elevated β-hCG levels, further supporting the biological relationship between trophoblastic activity and thyroid function [15]. Although clinical thyrotoxicosis has become less common because of earlier diagnosis, biochemical thyroid dysfunction may still occur in patients with molar pregnancy, particularly in those with complete mole and markedly elevated β-hCG concentrations. However, because the present study did not perform multivariable adjustment for potential confounding factors, including gestational age, maternal age, gravidity, and parity, these findings should be interpreted as associations rather than independent effects of β-hCG on thyroid function.

Strengths and limitations

The present study has several strengths, including its prospective design, histopathological confirmation of all cases, comprehensive assessment of thyroid hormone profile along with serum β-hCG concentrations, and direct comparison between complete and partial hydatidiform mole. However, certain limitations should be acknowledged. The study was conducted at a single tertiary care center with a relatively modest sample size, which may limit the generalizability of the findings. In addition, multivariable analysis was not performed to adjust for potential confounding factors such as gestational age, maternal age, gravidity, and parity; therefore, the observed associations between serum β-hCG and thyroid hormone parameters may be influenced by these factors. Karyotyping or molecular/ploidy analysis was not performed; therefore, the classification of complete and partial hydatidiform mole was based on the available clinical, ultrasonographic, and histopathological findings. Serial post-evacuation assessment of thyroid function and long-term follow-up for persistent gestational trophoblastic disease were also not performed. Larger multicentric studies with longitudinal follow-up, molecular/ploidy confirmation, and adjustment for potential confounders are warranted to further validate the observed relationship between serum β-hCG concentrations and thyroid dysfunction in molar pregnancy.

## Conclusions

Complete hydatidiform mole was associated with significantly higher serum β-hCG concentrations and more pronounced thyroid dysfunction than partial hydatidiform mole, characterized by lower TSH and higher free and total thyroid hormone levels. Serum β-hCG concentrations demonstrated a significant negative correlation with TSH and positive correlations with free and total T3 and T4 levels, supporting a close association between trophoblastic activity and thyroid function. These findings suggest that thyroid dysfunction may be particularly relevant in women with complete hydatidiform mole or markedly elevated serum β-hCG concentrations; however, the observed associations should be interpreted cautiously because potential confounding factors were not adjusted for in the present study. Larger multicentric studies with longitudinal follow-up are warranted to further clarify the clinical significance of the relationship between serum β-hCG concentrations and thyroid function in molar pregnancy.

## References

[1] Lok C, Frijstein M, van Trommel N (2021). Clinical presentation and diagnosis of gestational trophoblastic disease. Best Pract Res Clin Obstet Gynaecol.

[2] Nicheperovich A, Schuster-Böckler B, Ní Leathlobhair M (2025). Gestational trophoblastic disease: understanding the molecular mechanisms of placental tumours. Dis Model Mech.

[3] Dhanda S, Ramani S, Thakur M (2014). Gestational trophoblastic disease: a multimodality imaging approach with impact on diagnosis and management. Radiol Res Pract.

[4] Jagtap SV, Aher V, Gadhiya S, Jagtap SS (2017). Gestational trophoblastic disease - clinicopathological study at tertiary care hospital. J Clin Diagn Res.

[5] Yuk JS, Baek JC, Park JE, Jo HC, Park JK, Cho IA (2019). Incidence of gestational trophoblastic disease in South Korea: a longitudinal, population-based study. PeerJ.

[6] Assefa EM, Kassaw AB, Belete M (2025). Magnitude and histopathological patterns of gestational trophoblastic disease in Africa: a systematic review and meta-analysis. BMJ Open.

[7] Patil BU, Gangane NM, Shivkumar VB (2020). The frequency of hydatidiform mole in a tertiary care hospital from central India. Indian J Pathol Oncol.

[8] Braga A, Coutinho L, Chagas M (2025). Molar pregnancy: early diagnosis, clinical management, and the role of referral centers. Diagnostics (Basel).

[9] Stevens FT, Katzorke N, Tempfer C (2015). Gestational trophoblastic disorders: an update in 2015. Geburtshilfe Frauenheilkd.

[10] Nwabuobi C, Arlier S, Schatz F, Guzeloglu-Kayisli O, Lockwood CJ, Kayisli UA (2017). hCG: biological functions and clinical applications. Int J Mol Sci.

[11] De Guzman E, Shakeel H, Jain R (2021). Thyrotoxicosis: a rare presentation of molar pregnancy. BMJ Case Rep.

[12] Düğeroğlu H, Özgenoğlu M (2019). Thyroid function among women with gestational trophoblastic diseases. A cross-sectional study. Sao Paulo Med J.

[13] Shaaban AM, Rezvani M, Haroun RR (2017). Gestational trophoblastic disease: clinical and imaging features. Radiographics.

[14] Soper JT (2021). Gestational trophoblastic disease: current evaluation and management. Obstet Gynecol.

[15] Badlaeva A, Tregubova A, Asaturova A, Melli B, Cusenza VY, Palicelli A (2025). Hyperthyroidism associated with gestational trophoblastic neoplasia: systematic literature review and pathways analysis. Cancers (Basel).

[16] Zheng H, Wang Q, Chen F (2023). Correlation between serum beta-human chorionic gonadotropin levels and thyroid metabolic function in pregnant women with hyperemesis gravidarum. Chin J Physiol.

